# Intelligent Big Information Retrieval of Smart Library Based on Graph Neural Network (GNN) Algorithm

**DOI:** 10.1155/2022/1475069

**Published:** 2022-07-13

**Authors:** Lu Pang

**Affiliations:** Shandong Women's University, Jinan, Shandong 250014, China

## Abstract

In order to provide users with more humanized and intelligent big data knowledge services, a research method of intelligent big information retrieval of Smart Library Based on graph neural network (GNN) algorithm is proposed. Through the key technical problems and solutions of information recommendation represented by graph neural network (GNN) algorithm, this method explores how the library can realize the management and value mining of big data knowledge services. The research shows that the intelligent information retrieval of Smart Library Based on graph neural network (GNN) algorithm is 80% higher than the previous general methods. Graph neural network is a more advantageous algorithm for node classification, link prediction, node clustering, or network visualization, which is of great help to improve the efficiency of information retrieval.

## 1. Introduction

In today's era of rapid development of network, library still plays an important role in providing people with learning resources, carrying out academic research, and providing ways of knowledge acquisition. Especially for colleges and universities, library can provide students with rich learning resources and provide a strong knowledge system for scientific research, academic research, and network information services. With the continuous development of network technology, especially the emergence of cloud computing and big data technology, the traditional library must make timely adjustments, especially in the field of information retrieval, to pursue a more intelligent breakthrough and development. Make full use of big data analysis technology to build a modern, digital, and intelligent library. Therefore, combined with the needs of the construction of Smart Library and based on graph neural network algorithm, this paper proposes an automatic encoder system of graph neural network algorithm based on motif to better realize the intelligent big information retrieval of Smart Library [1]. Foreign scholars mainly study the principles, functions, methods, service objects, and evaluation direction of library information service. In the environment of the emergence and continuous development of the Internet and digital resources, we must reposition the library, measure it from what the library should do to what reason to guide the library to do, and lead to the change of the library service principle is the service strategy [2]. The domestic attempts to the library information service are mainly focused on the digital environment. The trend of library information service in the twenty-first century should be: openness, comprehensiveness, integration and efficiency, diversification, and personalization of information service. The purpose of library information service is to provide services for the public. At the same time, the role of library should help readers obtain the required information resources and be recognized by everyone.

Information service is a core business of the library, an important tool for the library to carry out subject knowledge service, and an important “bridge” for the knowledge exchange and communication between professional librarians and readers. For libraries that follow the traditional information consulting service model, whether consulting librarians can provide in-depth knowledge consulting services for readers often depends on whether they have a wide range of knowledge and good communication skills. Due to the different knowledge structure, literacy and professional skills of different consulting librarians, it often leads to problems such as uneven consulting service quality or superficial consulting. In the process of traditional consulting information analysis, readers rely more on the subjective industry experience of consulting experts. Facing the increasingly complex consulting situation, they are also easy to fall into the dilemma that the consulting effect deviates from the actual requirements.

Facing the challenges of the times, in order to improve the real-time and convenience of information consultation, the library is actively developing a distributed virtual information consultation system. The integrated consultation platform can give full play to the intellectual labor of subject librarians, provide users with a combination of real-time consultation and delayed consultation, and provide deep-seated, multiapplication scenario flexible switching services, so as to make up for the deficiency of delayed consultation under the traditional mode. Different from the “receiving service” of traditional consultation, some libraries build virtual knowledge platforms, create knowledge by answering questions, and provide knowledge services through mutual discussion among users [3]. The main role of the consulting librarian is to answer the user's questions into reviewing the questions or answers submitted by the readers, push the appropriate content to the users in a timely and targeted manner, or invite other users and experts to help answer. In addition, librarians undertake the task of transforming the tacit knowledge of librarians and users into explicit knowledge, and accumulate the answers to questions into the knowledge base, so as to provide reference for readers' future learning and research. At the same time, intelligent robot librarians also began to work. Service innovation is the only way to form the core competitiveness of library information consulting business. The traditional library consulting service is gradually moving towards a new generation of intelligent service mode [4].

## 2. Literature Review

Gao et al. proposed to establish a smart library system based on wireless sensor technology. Wireless sensor technology has the characteristics of automatic updating, adding or deleting publications, automatic guidance, and project retention mechanism [5]. Zhang mentioned that as a place to provide information services, the library also needs to gather knowledge to provide personalized knowledge services for users. Such complex and cumbersome work requires the development of a next-generation information service system based on the latest information and communication technology, that is, smart library. The smart library must add the knowledge-based online learning system to the original library service function to expand the number of people using the library. It must establish a collective and interactive page for readers to develop their collective wisdom consciousness and promote readers' creative thinking and logical thinking [6]. Bassey et al. also pointed out that nowadays, most library systems use client/server and ASP to serve their software. These services are usually difficult to manage and costly. So, he tried to build and implement the intelligent library integrated management system. He designed the system from three aspects: system architecture, concept map service, and development environment, discussed the feasibility of the system from the development scope, service scheme and multitenant environment, and finally analyzed the performance of the system [7]. Wang mentioned: if we compare the public reading room with the smart library, the Smart Library undoubtedly has great advantages in human resources, capital investment, and library opening hours. Some readers are unwilling to ask the librarian for help even if they encounter difficulties [8]. Wang et al. pointed out that many knowledge services have developed a matching platform for knowledge demanders and knowledge providers. However, most of these knowledge services have a common disadvantage, that is, they cannot provide experts with immediate services for knowledge demanders [8]. Swanson et al. pointed out that some foreign scholars believe that knowledge services mainly include four types: content, product, service, and scheme; Develop products including databases, technical reports, scientific papers, publicity materials, policies, rules, and information systems; Provide services, including lectures, answers, and suggestions; Share solutions, including planning, direction, attitude, and integration [9]. Kuftinova et al. mentioned that there are three types of knowledge services: one is literature retrieval; Second, focus on providing users with knowledge to meet their tasks; Third, share the knowledge required for similar work tasks among different people [10]. To sum up, smart library is the development trend of traditional library in the future. Although Smart Library is still in the primary stage in China and even in the world, it can be seen that the research progress in China is slightly backward compared with that in foreign countries, and most of them only stay in theoretical research. Chinese scholars should learn more from foreign research results and open up effective ways suitable for the development of China's smart library, so as to reshape the value and position of library knowledge service field [11].

## 3. Method

### 3.1. Intelligent Information Service Ecosystem Driven by Big Data

**Figure 1:**
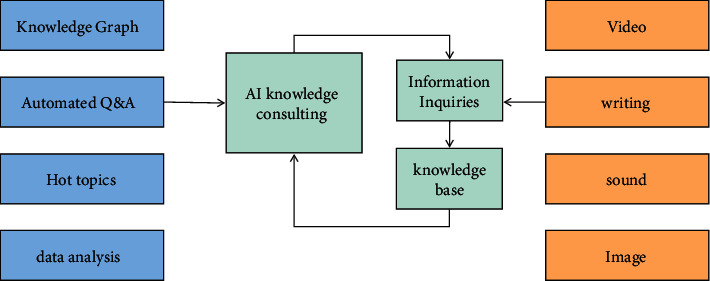
Closed-loop ecology of intelligent information consulting service.

In the information service platform, the knowledge exchange between consulting librarians and readers is easy to produce valuable interactive data, including all kinds of original video, sound, picture, text, and other information content. Artificial intelligence is associated with big data. The network robot with “smart algorithm” can automatically capture and exchange updated information in real time. Any reading behavior of users will be recorded truthfully, such as the number of clicks, the length of content reading, reader comments, forwarding, or sharing actions. The intelligent system carries out data cleaning, screening, mining, and analysis on these interactive data, semantically understands the user's questions, big data analyzes the user's reading interest, depicts a user's interest map, and then intelligently selects information according to the user's interest, and selects the appropriate application scenario to couple the best matching knowledge information with the target user. Finally, start the voice automatic answer to truly reflect the “information path” function of intelligent knowledge consultation [12]. Based on big data, with artificial intelligence as the core and with the goal of providing personalized, accurate and efficient information services, the new generation of intelligent knowledge consulting services form a closed-loop ecosystem of standardized and professional knowledge consulting services driven by big data, as shown in Figure 1.

**Figure 2:**
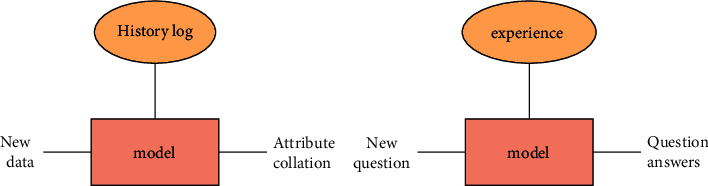
Automatic knowledge Q and a service framework.

With the advent of the era of big data, deep learning technology has broad application prospects in the fields of speech recognition, search recommendation, natural language processing, and so on. Libraries also gradually integrate deep learning, build intelligent knowledge automatic question and answer platform and carry out virtual consulting services [13]. Firstly, the intelligent consulting system continuously transmits the data to the cloud, compares these data with the algorithm of artificial intelligence, and applies “speech recognition” to automatically convert the recorded content generated by the user into the corresponding text or command. The emergence of cloud computing makes the centralized data computing and processing ability unprecedentedly powerful. Machines use deep learning technology for semantic recognition to complete content understanding and value judgment or convert into commands [14]. The “consulting brain” based on big data will associate the emerging content with the existing data for technical calculation, accurately understand the semantics for retrieval, and finally convert the output result into machine synthesized speech, and feedback the result to the user in the form of dialogue, as shown in Figure 2. It is difficult for the consulting librarian to input questions that are not defined in advance, but for the machine, as long as certain rules are met, the question and answer system can select the most relevant answer from a series of possible answers, which makes the reader feel like asking and answering with people rather than with the machine.

Different from the traditional consulting methods based on knowledge database retrieval, the end-to-end question answering system based on deep learning algorithm organically organizes important artificial intelligence technologies such as speech recognition, semantic understanding and speech synthesis, realizes the function of automatic question answering, and actively provides the subject knowledge associated with the problem. Intelligent knowledge consultation integrates deep learning, automatically extracts multilayer feature representation from massive low-level interactive data, from concrete to abstract, from general to specific, and extracts more complex features from simple features [15]. The goal is to make the machine not only recognize the text, image, sound, and other data submitted by readers, but also have the ability of analysis and learning like people, and use these combined features to help readers answer questions.

**Figure 3:**
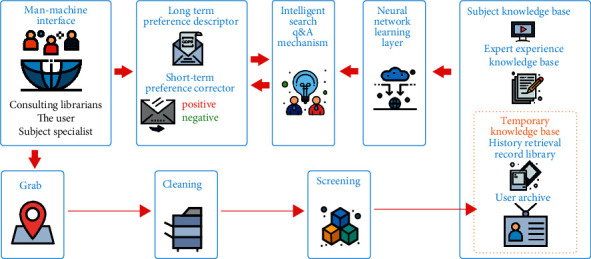
Neural network learning model of intelligent knowledge consulting platform.

The learning model of automatic question answering system based on deep learning simulates the learning ability of human brain, which depends on the neural network with multiple hidden layers. The hidden layer nodes of neural network generally exceed 5 layers, sometimes up to 10 layers. The learning model first allows each layer to learn the knowledge characteristics from the data source in advance, and then produces a series of valuable knowledge rules that have not been found in advance through the learning reasoning of the neural network learning layer [16]. The learning of neural network learning layer starts from the shallow layer in sequence, and gradually learns from the primary features of the shallow layer to the advanced features of the deep layer. The output data obtained from the learning of the upper layer will be used as the input data of the next layer. Finally, the initial sample data is transformed from the original feature space to a new feature space, and the joint distribution between input questions and output answers is established to achieve the purpose of feature learning of large-scale corpus data, which can make it easier to classify consulting questions or automatically predict question answers and improve the accuracy, as shown in Figure 3. The learning model emphasizes knowledge feedback learning and adopts the form of parameter weight to more effectively control the variable factors of the learning algorithm, so as to more actively adapt to the learning differences between new and old users [17].

The development of emerging technologies in the Internet and information technology industry has a great impact on the development of modern library information service mode. It brings the user-centered service concept to the library community, and makes the library regain its due position in the field of information and information. After the “library”, the concept of “smart Earth” was put forward in the field of global information technology at the end of the year. Inspired by it, on the basis of “library” and combined with the concept of “smart Earth”, the library community has developed the library information service mode to a more intelligent direction, and put forward the idea of “Smart Library”, which realizes the interaction between resources, users and applications through the Internet of things, and improves the efficiency of resource utilization. This paper puts forward “intelligent information service model based on Library”. This model adheres to the tenet of “user-centered and personalized service”, based on the construction of University Libraries and intelligent information services in line with the actual needs of libraries. Through “library intelligent information service”, under the condition of limited technology and economy of the library, we can also realize the progress towards more intelligent information service.

### 3.2. Graph Automatic Encoder Based on Motif Graph Neural Network Algorithm

#### 3.2.1. First and Second-Order Similarity

Redefine the first-order similarity. If two nodes exist in a motif at the same time, the two nodes have first-order similarity. The size of first-order similarity is defined as the number of motifs that exist together. The new first-order similarity includes the previous first-order similarity. If the motif type considered is *M*_21_, the two similarities are equal [18]. The new first-order similarity can exist between connected nodes or between nonconnected nodes. For example, in *M*_43_, any connected node and two pairs of diagonal nodes have first-order similarity. In this paper, *w*_*ij*_ is used to represent the first-order similarity of nodes *v*_*i*_ and *v*_*j*_. If there is no first-order similarity between two nodes, *w* is set to 0.

Redefine second-order similarity. This study first defines the neighbors of nodes: if two nodes are in the same motif, the two nodes are neighbors to each other. The new second-order similarity is obtained by quantifying the neighbor similarity of two nodes. This study redefines the adjacency vector of nodes according to the new neighbor concept as:

(1) $$\begin{array}{c} x_{i}=\left[w_{i1};w_{i2};\ldots ;w_{\text{in}}\right], \end{array}$$

where *w*_*ij*_ is the first-order similarity of nodes *v*_*i*_ and *v*_*j*_ redefined in (1). Therefore, the similarity of vectors *x*_*i*_ and *x*_*j*_ can be used to represent the second-order similarity of *v*_*j*_ and *v*_*j*_.

#### 3.2.2. Algorithm Flow

In order to use motif for link prediction, this study first learns a characterization vector for each node through the redefined first-order and second-order similarity, and then predicts the edge through the similarity of the vector. A simple way is to directly use the second-order similarity, that is, the adjacency vector as the representation vector of the node. However, adjacency vectors are not only high-dimensional and sparse, but also cannot fuse first-order similarity. The automatic encoder is proved to be an effective nonlinear dimensionality reduction tool, which is suitable for complex data. Therefore, the proposed algorithm combines motif and graph automatic encoder to generate the representation vector of nodes. Firstly, the algorithm compresses the redefined adjacency vector of nodes into a low dimensional dense vector through automatic coding. Then, due to the first-order similarity between nodes in a motif, the algorithm adds a supervision information to the output vector of the encoder to reduce the distance between all node vectors in the same motif [19].

Assuming that the encoder has *K* layers, the representation vector learning process of node *v*_*i*_ is as follows:

(2) $$\begin{array}{c} y_{i}^{1}=\sigma \left(W_{1}x_{i}+B_{1}\right),\\ y_{i}^{k}=\sigma \left(W_{k}y_{i}^{k-1}+B_{k}\right),\;k=2,\ldots ,K, \end{array}$$

where *σ* is the activation function, *W*_*k*_ and *W*_*k*_ are the weight and bias at layer *k*, and *x*_*i*_ is the adjacency vector redefined by the node. The output *y*_*i*_^*k*^ of the encoder is set as the final characterization vector *y*_*i*_ of the node. Coding is usually a process of dimensionality reduction, while decoding is a process of dimensionality increase [20]. The decoder accepts the output of the encoder and restores the input of the encoder. The decoder and encoder have the same number of layers. The specific calculation process is as follows:

(3) $$\begin{array}{c} y_{i}^{1}=\sigma \left(W_{1}^{\prime }y_{i}+B_{1}^{\prime }\right),\\ y_{i}^{k}=\sigma \left(W_{k}^{\prime }y_{i}^{k-1}+B_{k}^{\prime }\right),\;k=2,\ldots ,K, \end{array}$$

where *W*_*k*_′ and *B*_*k*_′ are the weights and biases of the k-th layer of the decoder. The automatic encoder needs to minimize the difference between the input of the encoder and the output of the decoder. Since the input and in and out are vectors, the algorithm minimizes their Euclidean distance, that is,

(4) $$\begin{array}{c} {\left\|x_{i}-x_{i}^{\prime }\right\|}_{2}^{2}. \end{array}$$

In this paper, {*M*} is used to represent the set of all motifs on the network. At this time, the loss function is defined as

(5) $$\begin{array}{c} L_{2nd}=\sum_{M\in \left\{M\right\}}\sum_{i\in V_{M}}{\left\|x_{i}-x_{i}^{\prime }\right\|}_{2}^{2}. \end{array}$$

Due to the sparsity of the network, there are often more zero elements in the adjacency vector of nodes. Therefore, automatic encoders tend to reconstruct zero elements. In order to solve this problem, each nonzero element has a weight. The loss function is defined as follows:

(6) $$\begin{array}{c} L_{2nd}=\sum_{M\in \left\{M\right\}}\sum_{i\in V_{M}}{\left\|\left(x_{i}-x_{i}^{\prime }\right)⊙z_{i}\right\|}_{2}^{2}, \end{array}$$

where Θ represents the multiplication of elements in the vector. The low-dimensional vector learned from the adjacency vector only retains the second-order similarity of nodes [21]. In order to integrate the first-order similarity into the automatic encoder, the algorithm adds a supervision information in the output layer of the encoder, so that the nodes in the same motif have similar representation vectors. The loss function of first-order similarity is defined as

(7) $$\begin{array}{c} L_{1st}=\sum_{M\in \left\{M\right\}}\sum_{i,j\in V_{M},i\neq j}{\left\|y_{i}-y_{j}\right\|}_{2}^{2}. \end{array}$$

The above formula reflects that the more motifs two nodes share, the closer their characterization vectors are. The vectors learned by the automatic encoder through formula (6) and (7) can reflect the first-order and second-order similarity between nodes at the same time. However, the above first-order similarity loss function only considers the similarity of nodes in motif, so the model is unstable and it is easy to make the characterization vectors of all nodes similar. The algorithm adds a random negative node outside the motif [22]. The supervision information not only makes the vector representation of each node in the motif similar, but also distinguishes the representation vectors of all nodes and negative nodes in the motif. Assuming that the algorithm samples negative node *v*_*i*_, the loss function is defined as follows:

(8) $$\begin{array}{c} L_{\text{dis}}=\max\left(\lambda +\sum_{i,j\in V_{M},i\neq j}{\left\|y_{i}-y_{j}\right\|}_{2}^{2}-\mu \sum_{i\in V_{M}}{\left\|y_{i}-y_{j}\right\|}_{2}^{2},0\right), \end{array}$$

where *μ* is a balance parameter, which is used to balance the difference in the number of nodes between the first-term accumulation and the second-term accumulation. For the third-order motif, *μ* is set to 1 because the sum of the first term and the second term is the sum of three Euclidean distances. The algorithm redefines the loss function of first-order similarity as

(9) $$\begin{array}{c} L_{1st}=\sum_{M\in \left\{M\right\}}\max\left(\lambda +\sum_{i,j\in V_{M},i\neq j}{\left\|y_{i}-y_{j}\right\|}_{2}^{2}-\mu \sum_{i\in V_{M}}{\left\|y_{i}-y_{j}\right\|}_{2}^{2},0\right). \end{array}$$

In order to prevent over fitting of the model, the algorithm adds a regularization to all learning weights in the model:

(10) $$\begin{array}{c} L_{\text{reg}}=\sum_{k-1}^{K}\left({\left\|W_{k}\right\|}_{F}^{2}+{\left\|W_{k}^{\prime }\right\|}_{F}^{2}\right), \end{array}$$

where ‖·‖_*F*_^2^ represents Frobenius norm. The final loss function is defined as

(11) $$\begin{array}{c} L_{\text{reg}}=L_{2n\;d}+\alpha L_{1st}+\gamma L_{\text{reg}}. \end{array}$$

Among them, *α* and *y* are two super parameters, which control the first-order similarity and the weight of regularization, respectively. The algorithm trains the model parameters by optimizing the loss function (11) and back propagation. In order to make the model converge faster, the algorithm uses Adam optimization method. Firstly, the algorithm needs to input the network, motif type, super parameters, the size of each batch of data, and learning rate. According to the selected motif type, the algorithm finds all motifs and calculates the adjacency vector of nodes. Before training the model, the algorithm initializes the model parameters. In order to train the model faster and prevent the model from falling into local optimization, the algorithm can use DBN to pre train the model. When training the model, the algorithm randomly extracts part of the motif in each round, and then calculates the loss function and optimizes the model parameters. When the model converges, the algorithm stops training and finally outputs the representation vector of each node. The output vector can be used for link prediction [23].

## 4. System Composition

### 4.1. Intelligent Resource Processing System

Intelligent resource processing system is the most basic application of library intelligent information service system. The purpose of intelligent resource processing system is to realize the digital processing and management of information resources. The digital processing of printed documents in the intelligent resource processing system generally adopts a structured network system, which integrates data processing, content management, and network publishing. The main process is as follows: the first step is to scan and digitize the printed documents, the second step is to recognize and proofread the scanned electronic documents, the third step is to process and digitize the proofread electronic documents, and the fourth step is to complete the digital storage of the printed documents and publish them to the intelligent resource storage system.  Table 1 shows the comparison between compression technology and output format of main digital processing systems.

### 4.2. Intelligent Resource Storage System

Intelligent resource storage system and intelligent resource processing system form the bottom data storage system of library intelligent information service system. Smart resource storage system provides users with stable and reliable physical facilities and strategic link guarantee for obtaining and utilizing information resource services. The intelligent resource storage system selects an appropriate digital resource storage system to store and save the information resources processed by the intelligent resource processing system. In the actual construction of the library, because the needs and funds of each library are different, it is difficult to reach the goal in one step, regardless of the size of the library. Therefore, in different libraries or information, according to the total amount of resources, the number of concurrent users and the status of capital investment, we need to choose a plan that not only meets the needs of library construction, but also has a certain forward-looking.  Table 2 shows four representative storage system schemes in the library intelligent information resource storage system.

### 4.3. Intelligent Resource Collection and Integration System

The intelligent resource collection and integration system provide an effective tool for the library intelligent information service system to search and integrate network resources and integrate them into the whole intelligent information service system. Intelligent resource collection and integration system is a system that collects and integrates the disordered network information resources distributed in the network through various intelligent technical means. Information integration refers to a service mode that collects information resources on the Internet and enables readers and users to find or browse relevant information resources through a unified retrieval platform after evaluation, classification, indexing, database building, and other processing. This has also become one of the important contents of the construction and service of information resources in university libraries [24]. At present, there are also some systems with network information resource integration in the picture information market, such as Tongfang's system and Beijing Tuoersi's network information radar system. However, it has not been widely used because of poor search accuracy, low coverage, and high price.

The network information source provided by the user is collected by the network information collection, and stored in the user interest model base according to the user classification. The network information is indexed by the information indexing, and stored in the indexed information base. The network information or indexed information is classified by the information classification. The intelligent information resource search and integration system with multicontrol reflect the intelligence, cooperation, and flexibility that the traditional integration system does not have, can make up for the problem of giving information sources in most existing integration systems, and can improve the quality of users' access to information resources and the efficiency of applying information resources.

### 4.4. Intelligent-Integrated Service System

Intelligent-integrated service system is an important part of intelligent information service system. It can effectively integrate all kinds of information and improve the quality of library information service. Intelligent-integrated service system is a new way to collect and express information resources with users as the core and personalized selection as the interface. It concentrates various services in the intelligent information service system on one platform, such as the integration of heterogeneous digital information resources and the establishment of unified retrieval. A basic idea for the construction of intelligent information integration service system based on Web services is to use WSDL to describe various digital resources and retrieval services of intelligent information service system, including service content, operation type, request and response message flow, and system binding mode. Then, the generated description information, that is, the metadata about the service system, is registered in the network service public registration system UDDI, and the service call between the service provider and the service consumer is established through UDDI, so as to realize the integrated information service.

### 4.5. Intelligent Service Collaboration System

When users encounter resource problems or service problems that cannot be solved when using the library intelligent information service system, the system will seek service assistance from other information service systems through the intelligent information service cooperation system. At present, this kind of document inspection and delivery service is mainly realized through agent inspection. Intelligent service collaboration system is a tool for information service collaboration between intelligent information service systems. When the user's information needs or service needs cannot be met in the intelligent information service system, the user can submit these information needs and service needs to the information service cooperation system of the intelligent service system. According to the detailed information provided by the user, the intelligent service cooperation system will send the information service cooperation request to other digital information service systems or special document delivery institutions that establish a contractual relationship for inspection and inspection, and feedback the results to the service cooperation system of the requesting party through online document delivery, and the service cooperation system of the requesting party will send the results to the end user in time.

### 4.6. System Structure Construction

**Figure 4:**
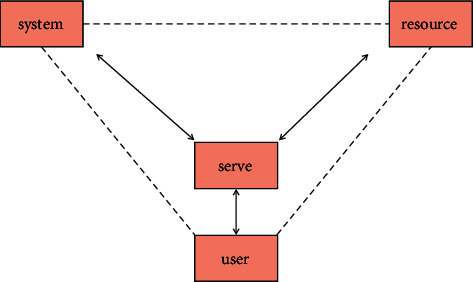
Service network structure of users, systems, and resources.

There are three typical management modes of library information service system, namely point management mode, linear management mode and network management mode, as shown in Figure 4.

**Figure 5:**
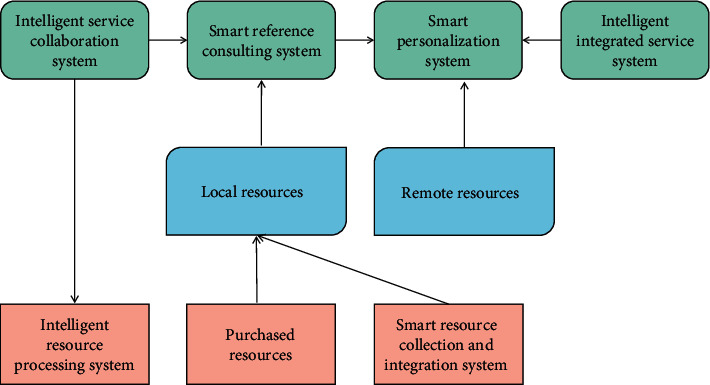
System composition and user relationship.

The architecture construction of library intelligent information service system should adopt the network management mode. Figure 4 shows the network architecture established through services between users, systems, and resources. The structure takes service as the starting point and establishes various services between the system, resources, and users, so as to interact between the system and resources, users, and resources. At the same time, it strengthens the understanding between users and resources, users and the system, so that users can assist librarians to participate in the construction of the library and avoid the blind development of the system separated from the actual needs of users. The system composition and user relationship diagram of the specific intelligent information service system are shown in Figure 5.

Intelligent information service system can be divided into resource layer and service layer. The resource layer of intelligent information service system is formed by intelligent resource processing system, intelligent resource storage system, and intelligent resource collection and integration system, and the service layer of intelligent information service system is formed by intelligent service cooperation system, intelligent service integration system, intelligent personalized service system, and intelligent reference system.

**Figure 6:**
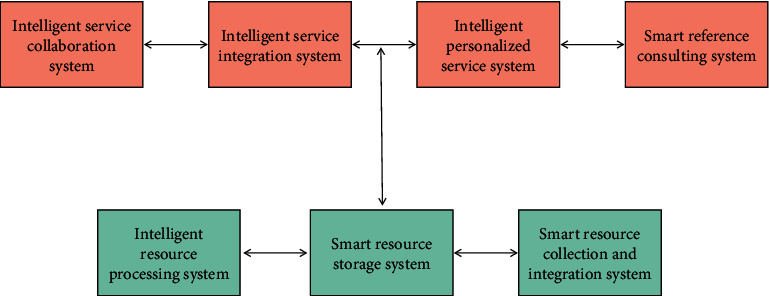
Construction mode of library intelligent information service system.

The ideal model for the construction of the library intelligent information service system is to include all the elements of the intelligent information service system, so as to provide users with various services such as digital information resource processing, information storage, resource collection, network resource integration, information integration, virtual consultation, and so on, as shown in Figure 6:

The service layer directly faces users through the library portal. When users have information service needs, a system of the service layer accepts the service needs of users, and feeds back the resource information to users through the resource layer of the intelligent information service system to complete the information service operation. At the same time, the intelligent reference system and intelligent resource collection and integration system record the user's use log when providing information services for users, so as to provide user data for the intelligent personalized service system. When users encounter problems that cannot be solved by the system itself when using the intelligent information service system, the system librarian will seek help from other libraries or information and intelligence institutions through the intelligent service cooperation system, and provide users with the greatest help as much as possible through agent inspection, document delivery, and other means. The nondigital resources transmitted by other libraries or information and intelligence institutions will be transferred to the intelligent resource processing system for digital processing, and then participate in the cycle of the intelligent information service system.

## 5. Experimental Results

### 5.1. Data Set

In this study, six public network data sets were used for experiments. See Table 3, for detailed data set statistics.

YouTube and LiveJournal are two social networks. A node represents a user, and an edge represents the friendship between the corresponding two users. In the original Youtube and LiveJournal data sets, 5246 nodes and 2456 nodes were randomly selected, and the connectivity of the whole network was guaranteed.

Bio-sc-cc and Bio-sc-ht are two biological networks. A node represents a gene, and an edge represents that there is an interactive relationship between the two corresponding genes. Bio-sc-cc consists of 2236 nodes and 34879 edges. Bio-sc-ht consists of 2084 nodes and 63027 edges.

DBLP and Ca-GrQc are two academic networks. A node represents a scholar, and an edge represents a cooperative relationship between the corresponding two scholars. In the original DBLP and Ca-GrQc data sets, 4244 and 4158 nodes were randomly selected in the experiment, and the connectivity of the whole network was guaranteed.

### 5.2. Experimental Setup

#### 5.2.1. Traditional Methods

(1) Common Neighbors(CN) define node similarity:
  (12) $$\begin{array}{c} \left|N_{i}\cap N_{j}\right|. \end{array}$$
(2) Adamic Adar(AA) their similarity is defined as
  (13) $$\begin{array}{c} \frac{\left|N_{i}\cap N_{j}\right|}{{\left|N_{i}\cap N_{j}\right|}^{\circ }}. \end{array}$$
(3) Jaccard Coefficient(JC) their similarity is defined as

(14) $$\begin{array}{c} \frac{\sum_{t\in N_{i}\cap N_{j}}1}{\log\;{\left|N_{t}\right|}^{\circ }}. \end{array}$$

The method is based on graph neural network:

1. SDNE uses the original first-order and second-order similarity. It inputs the adjacency vector of nodes into the automatic encoder and uses the first-order similarity as the supervision information.
2. Scat uses Gaussian transformation as encoder and full-link network as decoder. The decoder can be used as link prediction.
3. RGCN uses the coder of the drawing machine to learn the representation vector of the node. Its decoder uses tensor decomposition to predict the label of the edge.

**Figure 7:**
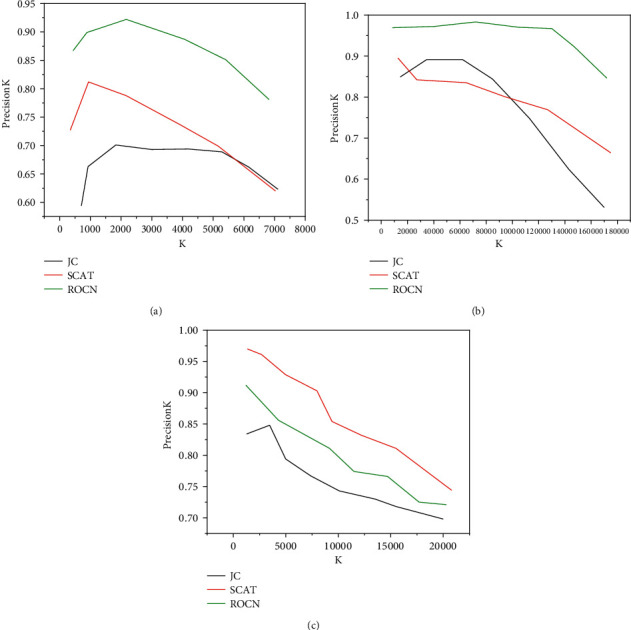
Comparison of ten algorithms on PrecisionK index. (a) Youtube. (b) LiveJournal. (c) Bio-sc-cc.

Table 4 shows the results on AUC, in which the best effect is bold. This table shows that the algorithm model is better than other methods in most cases.  Figure 7 shows the results of all algorithms on PrecisionK. The figure shows that as the number of predicted edges increases, the advantages of model are gradually increasing. On the social network Youtube, model is at least 20.6% ahead of traditional methods and at least 14.4% ahead of Web-based embedding methods. The method based on graph neural network is at least 3.1%. On the social network LiveJournal, the result of model is only slightly lower than that of scat, but it is largely ahead of other methods, including two graph neural network methods SDNE and RGCN.  Figure 7(a) and 7(b) shows that with the increase of K, the proportion of model leading these methods is also increasing.  Figure 7(c) shows in social networks, although hone also considered motif, it achieved the worst experimental results. On the LiveJournal data set, hone only achieved a result of 0.535. When the comparison is small, hone can achieve better results. However, when *k* increases, the result of hone decreases sharply, so it can only predict some edges.

**Figure 8:**
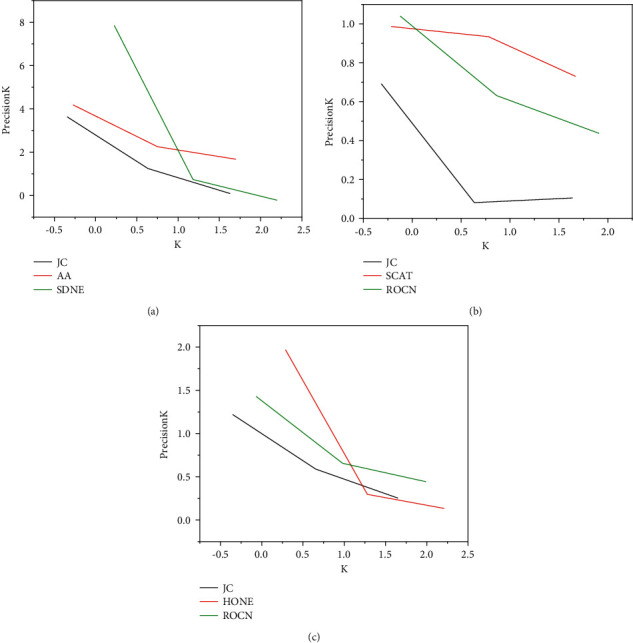
Avg. rank for predicting weak connections. (a) Bio-sc-ht. (b) DBLP. (c) Youtube.

Figure 8 shows the results of all algorithms in predicting edges with a number of common neighbors less than or equal to 2. When the number of shared neighbors is zero, the three traditional methods CN, JC, and AA achieve the worst results on these six data set networks. This shows that these three methods cannot accurately predict weak connections. The algorithm model has achieved good results on these data sets, and achieved the best results on Bio-sc-ht, DBLP, and Ca-Gr Qc. However, when the number of shared neighbors is 1 or 2, the traditional method achieves the best results.

Figure 8(a)–8(c) shows these two similarities are applicable to a variety of network analysis tasks. The algorithm model integrates the first-order and second-order similarity through the automatic encoder, learns a representation vector for each node, and uses the negative sampling optimization model. The experiment of parameter sensitivity shows the importance of balancing the first-order and second-order similarity, and better results can be obtained by selecting higher dimensional vectors. Further experiments show that the model algorithm has low time complexity.

## 6. Conclusion

By discussing the development of university libraries, this paper points out that most modern university libraries will exist in the form of compound libraries. In the research on the information service and information service system of university library, the author analyzes and compares the characteristics of library information service in each stage. At present, the resource needs of university library users have changed greatly. The information service of University Library presents the characteristics of diversification, personalization, specialization, integration, and interaction. The existing information service mode of university library cannot provide cross database one-stop retrieval, lack of integrated personalized service and compound reference service, and lack of interactive collaborative construction system, which has seriously affected the development of university library. This paper gives the construction and composition of the library intelligent information service system, and introduces the library intelligent information resource processing system, the library intelligent information resource storage system, the library intelligent information resource collection and integration system, the library information personalized service system, the library intelligent reference system, and the library intelligent information service cooperation system. This paper analyzes the problems and deficiencies of the existing information service system in information resource processing, storage, integration, and personalized service, and gives the corresponding solutions.

## Figures and Tables

**Table 1:** Comparison between compression technology and output format of main digital processing systems.

Digital processing system	Compression format	Output format
TPI	The binary image is compressed by JBIG and the gray image is compressed by JPEG	CAJ,PDF,KDH
TBS	G4 compression is used for black-and-white images, and JPEG and JPEG2000 compression are used for gray and color images	PDF
TRS	The text is compressed by LZW	PDF,HTML,XML

**Table 2:** Four storage system schemes of library intelligent information resource storage.

Capacity and requirements	Library type	Applicable storage system structure
When the digital capacity is only and TB	Small and medium-sized libraries with one-time investment	NAS storage system architecture
When the digital capacity is greater than 10 TB	Library with an increasing amount of data	SAN storage system architecture
When the digital capacity is large but the number of concurrent users is not too large	Library with the fastest growth of data volume	IPSAN storage system architecture
When the digital capacity is large and the number of concurrent users is large	Large library and information data center	NAS and SAN converged architecture

**Table 3:** Statistics of six data sets.

Data set	Number of nodes	Number of edges	Average node degree	Network type
Youtube	5145	24121	8.02	Social networks
LiveJournal	2435	18490	154.49	Social networks
Bio-sc-cc	2126	35279	31.2	Biological network
Bio-sc-ht	2075	52027	60.59	Biological network
DBLP	4424	12168	5.5	Academic network
Ca-GrQc	4158	15422	6.46	Academic network

**Table 4:** Comparison of ten algorithms in AUC index.

Algorithm	Youtube	LiveJournal	Bio-sc-cc	Bio-sc-ht	DBLP	Ca-GrQc
MODEL	0.623	0.819	0.793	0.842	0.805	0.820
DeepWalk	0.368	0.704	0.677	0.654	0.835	0.662
Node2vec	0.784	0.736	0.658	0.824	0.953	0.852
SDNE	0.632	0.738	0.725	0.841	0.821	0.805
HONE	0.724	0.520	0.406	0.756	0.801	0.781

## Data Availability

The labeled data set used to support the findings of this study is available from the author upon request.
